# Retrospective Cohort Study on the Efficacy and Safety of Endoscopic Submucosal Dissection for Gastrointestinal Lesions: A Single-Center Experience

**DOI:** 10.34172/mejdd.2025.440

**Published:** 2025-10-31

**Authors:** Pedram Beigvand, Seyed Hassan Abedi, Saman Alhooei

**Affiliations:** ^1^Department of Internal Medicine, School of Medicine, Babol University of Medical Sciences & Health Services, Babol, I.R.Iran

**Keywords:** Mass, Gastrointestinal, Resection, Endoscopy, En bloc

## Abstract

**Background:**

Gastrointestinal (GI) lesions, including precancerous and early-stage cancerous lesions, are a significant global health concern. Endoscopic submucosal dissection (ESD) has emerged as a minimally invasive treatment option, offering complete en bloc resection with lower recurrence rates compared with traditional surgical methods. However, the efficacy and safety of ESD, particularly in diverse clinical settings, require further evaluation. This study aimed to assess the outcomes of ESD for GI lesions in a single-center cohort, focusing on complete resection rates, complications, and recurrence.

**Methods:**

A retrospective cohort study was conducted at Ayatollah Rouhani Hospital in Babol, Iran, from 2018 to 2023. The study included 45 patients with GI lesions who underwent ESD, with 39 patients receiving en bloc resection and six undergoing piecemeal resection. Data on patient demographics, lesion characteristics, resection outcomes, and complications were collected. Follow-up endoscopic examinations were performed every 3 to 6 months to monitor for recurrence or residual lesions. Statistical analysis was performed using SPSS software version 26, with descriptive statistics, *t*-tests, and chi-square tests used to evaluate outcomes.

**Results:**

The mean age of participants was 61.92 years (±11.19), with a male predominance (57.7%). Lesions were primarily located in the stomach (46.6%), rectum (26.6%), colon (20%), and esophagus (6.6%). The complete resection (R0) rate was 95.1%, with no significant differences based on lesion location. Complications included three cases of perforation (6.6%) and three cases of immediate bleeding (6.6%), all of which were managed endoscopically without surgical intervention. Recurrence occurred in two cases (4.4%), both in lesions larger than 2 cm. En bloc resection demonstrated lower recurrence (2.5%) and perforation rates (5.1%) compared with piecemeal resection (16.7% recurrence, 16.7% perforation).

**Conclusion:**

ESD is a safe and effective technique for the complete en bloc resection of GI lesions, with high success rates and low recurrence. En bloc resection is superior to piecemeal resection, particularly for larger lesions. While complications such as perforation and bleeding are rare, they can be managed endoscopically. These findings support the use of ESD as a viable alternative to surgery, especially for elderly patients or those with surgical contraindications. Further multicenter studies with long-term follow-up are needed to validate these results and optimize ESD techniques globally.

## Introduction

 For many years, surgical intervention was the only treatment for large colon tumors, and endoscopic mucosal resection (EMR) was mainly used for smaller lesions below 2 cm or segmental resection of larger lesions.^1^ Currently, contemporary practice has shifted towards endoscopic resection as the preferred method for managing precancerous lesions, dysplasia, and early-detected gastrointestinal cancers.^2^ In Japan, endoscopic resection accounts for approximately 60% of early gastric cancer treatments.^3^ Recent advancements have introduced various endoscopic resection techniques, ranging from simple polypectomy to EMR and the more recent endoscopic submucosal dissection (ESD).^4^ Initially developed in Japan for early-stage cancers, ESD techniques have been adapted for use throughout the gastrointestinal tract.^5^

 In the EMR procedure, a local injection of normal saline or sodium hyaluronate is administered into the submucosal layer of a superficial tumor. A snare is then positioned around the lesion, and resection is performed using high-frequency current.^6^ In cases of piecemeal EMR, larger nodules or carcinomatous areas are excised in multiple fragments. Additionally, a novel technique known as underwater EMR allows for resection without prior saline injection into the submucosal layer.^7^

 In contrast, the ESD method involves injecting normal saline or sodium hyaluronate into the submucosal layer, followed by a circular incision of the lesion with a needle-shaped instrument and electrocautery current.^6^ This technique enables the complete resection of the entire lesion in a single piece, regardless of its size.^6^ The advantages of ESD over EMR include the ability to remove the entire lesion regardless of its size, a lower recurrence rate, obtaining an ideal specimen for histopathological experiments, and the successful removal of lesions that cannot be resected by EMR.^8^ Additionally, compared with surgery, ESD is associated with a lower complication rate, a more prompt recovery, shorter hospitalization, and lower costs.^9^

 However, ESD is more complex and time-consuming than EMR, requires greater skill and expertise of the endoscopist, and carries a higher risk of complications.^10^ The primary objective of endoscopic treatment for colorectal carcinoma is to achieve en bloc resection.^11^ While piecemeal EMR may be acceptable for certain adenomas and carcinomas, it is crucial to avoid fragmentation to prevent further difficulties in pathological evaluations.^12^ Magnifying endoscopy can significantly reduce the recurrence rate by evaluating lesion margins and wound depth post-resection.^13^ For larger lesions, more than half of the colon lumen, ESD is recommended over piecemeal EMR, especially when performed by skilled endoscopists. Surgical intervention is considered an alternative approach only when ESD is unavailable.^11,14^

 Research indicates that ESD is more effective than EMR for lesion removal, particularly in cases with a positive non-lifting sign or recurrent/residual tumors.^2,6,7^ However, ESD demands more time, advanced technical skills, and carries an elevated risk of adverse events.^15^ When determining the appropriate endoscopic treatment, it is essential to consider the patient’s comorbidities and current medications, particularly for those on antithrombotic therapy, as these can lead to bleeding if not appropriately managed before the procedure.^1,9,16,17^

 Both ESD and EMR are considered high-risk procedures due to the potential for hemorrhage.^18^ Although recent advancements offer different techniques for ESD in colorectal cancers, it still remains complicated for upper gastrointestinal lesions owing to the risk of perforation.^19^ The incidence of perforation during ESD for gastric lesions ranges between 1.2% and 2.5%, which is contributed to the lesion’s location and size.^20^ Additional complications may include immediate and delayed hemorrhage, luminal stenosis, and the impact of anticoagulant and antiplatelet medications on bleeding possibility.^21^

 ESD is a practical option for large superficial colorectal tumors, offering a higher rate of en bloc resection and a less invasive procedure than surgery.^18^ However, surgery remains the standard treatment for lesions with deep submucosal carcinoma or evident invasive characteristics.^11,22^ Furthermore, surgery is recommended in cases where there is lymphovascular invasion, submucosal infiltration exceeding 1000 μm (SM1), a non-evaluable lateral or vertical margin (R1, Rx), or a poorly differentiated tumor with any grade of submucosal invasion. Despite the remarkable benefits of ESD in managing gastrointestinal lesions, these methods are not widely used globally, and in our country, this is due to their novelty, potential complications, and the lack of experienced endoscopists.^23^ ESD is not yet universally recommended as the gold standard for managing colorectal lesions, and in some countries, it is performed as an advanced medical treatment without national health insurance coverage.^24^ However, in the near future, colorectal ESD is expected to become a preferred treatment choice for early colorectal cancer.^25^

 The primary objective of this study was to evaluate the efficacy and safety of complete en bloc resection of gastrointestinal lesions utilizing the ESD technique. This study aimed to assess the success rate of en bloc resection, the incidence of complications, and the recurrence rate following ESD procedures. Through reviewing endoscopic resection cases performed at Ayatollah Rouhani Hospital in Babol, the study aimed to gain insights into the outcomes of ESD, with the intention of reducing the surgical rate and complications associated with this treatment. The findings aim to enhance the understanding of ESD as a viable treatment option for gastrointestinal lesions, thereby facilitating the recognition and adoption of ESD as a standard procedure in clinical practice.

###  Study Design and Methodology 

 This study employed a retrospective cohort design to evaluate the outcomes of patients who underwent ESD for gastrointestinal lesions.

###  Study Population and Sample

 The study population consisted of patients referred to the Endoscopy Department of Ayatollah Rouhani Hospital in Babol from 2018 to 2023. The sample included patients with precancerous and cancerous lesions of the gastrointestinal tract, selected over a 5-year period.

## Materials and Methods

 The following materials and instruments were utilized in this study.

- *Questionnaire*: A structured, pre-validated questionnaire was administered to collect demographic data, clinical characteristics, and procedural outcomes following endoscopic lesion resection. The questionnaire comprised sections on patient age, sex, lesion location, size, histopathological findings, and post-resection complications.
- *Endoscope/Colonoscope*: high-definition video endoscopes from the Olympus EVIS EXERA III series (model CV-190) were employed. This system features advanced imaging capabilities, including narrow-band imaging (NBI) and high-resolution white light endoscopy, which incredibly enhance lesion detection and characterization.
- *Electrocautery Device*: An Erbe VIO 300D electrosurgical generator was utilized to assure exact tissue dissection and hemostasis.
- *Endoscopic T-knife*: A dual knife J (KD-655Q) was employed for ESD. This insulated-tip knife facilitates controlled mucosal incision and submucosal dissection while minimizing the risk of perforation.
- *Snare*: Captivator II snares in various diameters (10-25 mm) were selected according to lesion size for en bloc or piecemeal resection.
- *Submucosal Injection Needle*: A 23-gauge FlexKnife Injection Needle was utilized to administer a submucosal lifting solution from Endoflex Company.

###  Inclusion and Exclusion Criteria

####  Inclusion Criteria

- Lesions must be accessible during gastrointestinal endoscopy.
- Absence of metastasis in malignant lesions.
- Absence of regional lymph node involvement.

####  Exclusion Criteria

- Pregnancy.
- Known coagulation disorders.
- Underlying diseases place patients in a high-risk group for surgery.

###  Sample Size Determination

 The sample size was estimated at 30 patients based on a success rate of 80% using a specific formula.


$$n=\frac{Z_{1-\frac{\alpha }{2}}^{2}\;p\left(1-p\right)}{d^{2}}\;n=\frac{1.96^{2}.0.8\left(0.2\right)}{0.15^{2}}=30$$

α = 0.05, p = 80%, d = 0.15, n = 30

###  Data Collection

 This retrospective cohort study spanned 6 years (2018-2023) and focused on patients undergoing ESD for gastrointestinal lesions at Ayatollah Rouhani Hospital. Patients were informed about standard surgical options and the alternative ESD treatment, providing informed consent upon choosing ESD. A structured questionnaire was used to gather demographic and medical information. Medical records were reviewed, and procedures were explained thoroughly. During ESD, resections were performed by medical students under supervision. Follow-up endoscopies were scheduled every 3 to 6 months post-discharge to monitor for recurrence or residual lesions.

###  Statistical Analysis

 Data from completed questionnaires were analyzed using SPSS software version 26. Descriptive statistics were presented in tables, graphs, ratios, and percentages. T-tests were used for quantitative variables, while chi-square and Fisher’s tests were used for qualitative variables. All analyses were conducted at a 95% confidence level, with a *P *value of less than 0.05 considered statistically significant.

## Results

**Figure 1:**
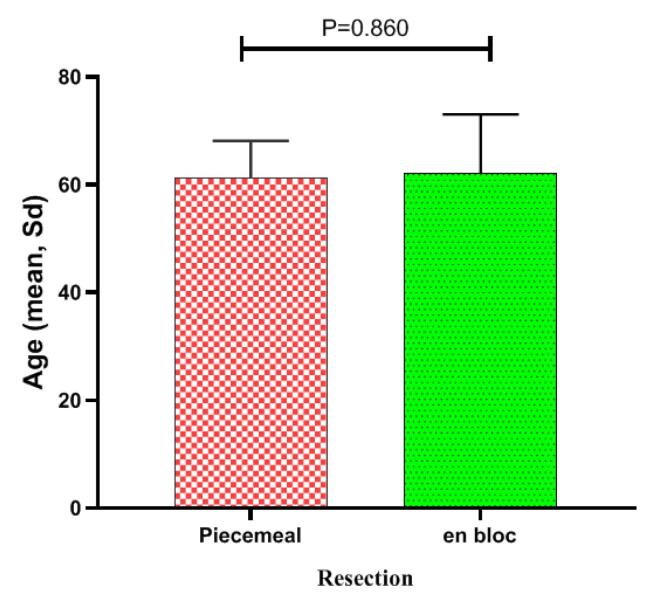
Mean and standard deviation of the age of patients with gastrointestinal lesions participating in the study by resection method

 In this study, 52 patients with gastrointestinal lesions who underwent complete en bloc resection using ESD in the endoscopy department of Ayatollah Rouhani Hospital in Babol during the years 2018-2023 were examined. Among these, seven patients were excluded from the study because they failed to return for follow-up care. Of the 45 patients examined, six underwent resection using the piecemeal method, and 39 underwent resection using the en bloc method. The mean age of the patients was 61.92 ± 11.19 years in total, and 61.17 ± 7.03 and 62.06 ± 11.89 years in the piecemeal and en bloc methods, respectively, as shown in Figure 1.

 According to the findings in Table 1, the en bloc method revealed a sex distribution of 53.8% male and 46.1% female. 46.1% were aged 60 years or less, and 53.8% were over 60 years old. In the piecemeal method, the sample consisted of 83.3% males and 17.7% females, with 33.3% aged 60 years or less and 66.7% over 60 years old. No significant difference was observed between the two lesion resection methods in terms of age and sex.

**Table 1:** Frequency of sex and age group of patients with gastrointestinal lesions participated in the study, by lesion resection method, and age group

**Variables**	**Resection method**		***P*** ** value**
	**En bloc** **No. (%)**	**Piecemeal** **No. (%)**	
Sex			
Male	21 (8.53)	5 (83.3)	197.0
Female	18 (1.46)	1 (17.7)	
Total	39 (100)	6 (100)	
Age			
60 years and under	18 (.146)	2 (33.3)	.9980
Above 60 years	21 (53.8)	4 (66.7)	
Total	39 (100)	6 (100)	

 Based on the findings in Table 2, out of 45 lesions, a total of 21 cases (46.6%) were gastric (fundus, antrum, greater curvature, lesser curvature, cardia), 12 cases (26.6%) were rectal, 9 cases (20%) were other parts of the colon (sigmoid, ascending colon), and 3 cases (6.6%) were esophageal.

**Table 2:** Frequency of anatomical location of gastrointestinal lesions in patients who participated in the study

**Anatomical location**	**Number**	**Percent**
Rectum	12	26.6
Sigmoid colon	5	11.1
Descending colon	1	2.2
Ascending colon	3	6.6
Esophagus	3	6.6
Stomach fundus	1	2.2
Stomach antrum	7	15.5
Greater flexure of the stomach	5	1.1
Lesser flexure of the stomach	5	11.1
Cardia	3	6.6
Total	45	100

 According to the findings in Table 3, no significant difference was observed between the anatomical location of gastrointestinal lesions and the method of lesion resection.

**Table 3:** Frequency of anatomical location of gastrointestinal lesions in patients who participated in the study by lesion resection method.

**Anatomical location**	**Resection method**		***P*** ** value**
	**En bloc** **No. (%)**	**Piecemeal** **No. (%)**	
Rectum	10 (222.)	2 (33.33)	0.640
Other parts of the colon	8 (17.7)	1 (16.67)	
Esophagus	2 (.66)	1 (16.67)	
Stomach	19 (2.42)	2 (3333.)	
Total	39 (100)	6 (100)	

 According to the findings in Table 4, three patients, including one man and one woman (in en bloc resection) and one woman (in piecemeal resection), suffered from perforation. In all three cases, the wall defect was repaired using hemoclips. The patients were only observed while receiving intravenous antibiotics, and in none of the cases did surgical repair become necessary. Additionally, bleeding occurred during the procedure in three cases, and in all of them, bleeding was stopped using Argon Plasma Coagulation (APC). There was no delayed bleeding in any of the patients. Additionally, in the follow-up after the procedure, two patients experienced recurrence of lesions (one patient in each en bloc or piecemeal method, and both patients were men). In one case, the lesion was low-grade dysplasia, and it was completely removed again with ESD. The second case in the study was rectal adenocarcinoma with stage T3N1, which was referred to the surgeon for resection. No significant difference was observed between the resection method and the complications of perforation and recurrence.

**Table 4:** Frequency of outcome of gastrointestinal lesion resection in patients participating in the study by lesion resection method

**Variable **	**Total** **No. (%)**	**Resection method**		***P*** ** value**
		**En bloc** **No. (%)**	**Piecemeal** **No. (%)**	
Perforation				
No	(93.3) 42	(94.8) 37	(83.5) 5	0.412
Yes	(6.6) 3	(5.1) 2	(16.7) 1	
Total	(100) 45	(100) 39	(100) 6	
Immediate bleeding				
No	(93.3) 42	(97.4) 38	(66.6) 4	0.568
Yes	(6.6) 3	(2.5) 1	(33.3) 2	
Total	(100) 45	(100) 39	(100) 6	
Delayed bleeding				
No	(100) 45	(100) 39	(100) 6	0.999
Yes	(0) 0	(0) 0	(0) 0	
Total	(100) 45	(100) 39	(100) 6	
Recurrence of the lesion				
No	(95.5) 43	(97.4) 38	(83.3) 5	0.294
Yes	(4.4) 2	(2.5) 1	(16.7) 1	
Total	(100) 45	(100) 39	(100) 6	

 Owing to the result, illustrated in Table 5, regarding pathology, T.V. adenoma + high-grade dysplasia (HGD), adenocarcinoma, and gastrointestinal stromal tumor (GIST) were the most common pathology findings with 26.6%, 22.2% and 15.5% respectively.

**Table 5:** Frequency of pathological findings of gastrointestinal lesions in patients participating in the study by lesion resection method

**Pathological findings**	**N**	**%**
Adenocarcinoma	10	22.2
Gastrointestinal stromal tumor	7	15.5
Heterotropic pancreas	1	2.2
Hyperplastic polyp	3	6.6
Juvenile polyp	1	2.2
Tubular adenoma with low-grade dysplasia	2	4.4
Neuroendocrine tumor	2	4.4
SCC	1	2.2
Sessile serrated adenoma with high-grade dysplasia	1	2.2
Tubulovillous adenoma with high-grade dysplasia	12	26.6
Brunner`s gland tumor	1	2.2
No pathology findings	4	8.8
Total	45	100

 A significant relationship was observed between the pathological results of gastrointestinal lesions according to the anatomical location of the lesion (*P* < 0.001, Table 6).

**Table 6:** Frequency of pathology findings of gastrointestinal lesions in patients who participated in the study by anatomical location of the lesion

**Pathological findings**	**Rectum** **% (N)**	**Other parts of the colon ** **(%) N**	**Esophagus** ** (%) N**	**Stomach ** **(%) N**	***P*** ** value**
Adenocarcinoma	50.5% (6)	0% (0)	0% (0)	21% (4)	< 0.001
GIST	0% (0)	0% (0)	0% (0)	36.8 % (7)	
Heterotropic pancreas	0% (0)	0% (0)	0% (0)	5.2% (1)	
Hyperplastic polyp	0% (0)	0% (0)	0% (0)	15.7 % (3)	
Juvenile polyp	8.3% (1)	0% (0)	0% (0)	0% (0)	
Tubular adenoma + LGD	0% (0)	0% (0)	0% (0)	10.5% (2)	
NET	8.3% (1)	0% (0)	0% (0)	5.2% (1)	
SCC	0% (0)	0% (0)	100% (1)	0% (0)	
SSA + HGD	0% (0)	11.1% (1)	0% (0)	0% (0)	
Tubulovillous adenoma + HGD	33.3% (4)	88.8% (8)	0% (0)	0% (0)	
Brunner`s gland tumor	0% (0)	0% (0)	0% (0)	5.2% (1)	
**Total**	100% (12)	100% (9)	100% (1)	100% (19)	

Abbreviations: GIST, Gastrointestinal stromal tumor; LGD, low grade dysplasia; HGD, high grade dysplasia; NET, neuroendocrine tumor; SCC, squamous cell carcinoma; SSA, sessile serrated adenoma.

Table 7 shows that the complete resection success rate was 95.1%. No significant relationship was found between the anatomical locations of gastrointestinal lesions in the patients who participated in the study, categorized by R0. Moreover, no significant relationship was observed between lesion size and the pathological results of gastrointestinal lesions.

**Table 7:** Frequency of anatomical location of gastrointestinal lesions in patients who participated in the study based on pathologist confirmation of R0 resection.

**Anatomical location **	**R0 resection**			***P*** ** value**
	**Yes ** **N (%)**	**No ** **N (%)**	**Total**	
Rectum	(100) 12	(0) 0	(100) 12	0.485
Other parts of the colon	(88.8) 8	(11.1) 1	(100) 9	
Esophagus	(100) 1	(0) 0	(100) 1	
Stomach	(94.7) 18	(52) 1	(100) 19	
Total	(95.1) 39	(4.9) 2	(100) 41	

## Discussion

 In this study, the average age of patients with gastrointestinal lesions was 61.92 years, with a male predominance (57.7% male vs. 42.3% female). These findings align with previous research indicating that gastrointestinal lesions are most commonly diagnosed during the seventh decade of life and are more prevalent in men.^26,27^ This sex disparity may be attributed to biological differences, lifestyle factors, or variations in early screening and diagnostic practices, particularly in regions like Iran, where the incidence of gastrointestinal tumors may occur at a younger age compared with Western populations.^27^

 ESD has emerged as the gold standard for treating gastrointestinal lesions due to its ability to provide continuous visual control during mucosal incision and its precision in distinguishing neoplastic from normal tissue.^28^ In our study, the recurrence rate following en bloc resection was 2.5%, with perforation and immediate bleeding rates of 5.1% and 2.5%, respectively. No cases of delayed bleeding were observed. These results are consistent with prior studies, which report recurrence rates ranging from 2% to 5% and perforation rates between 4% and 6% for ESD.^15,22^ The variability in complication rates can be influenced by factors such as tumor stage, lesion size, surgeon’s experience, and follow-up duration. For instance, lesions in the colon, where the wall is thinner compared with the stomach, are associated with higher perforation rates.^16^

 The complete resection (R0) rates in our study were 100% for rectal and esophageal lesions, 94.7% for gastric lesions, and 88.8% for colon lesions, yielding an overall R0 rate of 95.1%. These findings underscore the efficacy of ESD in achieving complete resection, which is critical for reducing recurrence rates.^29^ When comparing piecemeal and en bloc resection techniques, we observed higher perforation (16.7% vs. 5.1%) and recurrence (16.7% vs. 2.5%) rates with the piecemeal method, although no significant differences were noted in delayed bleeding rates. These results highlight the superiority of en bloc resection in minimizing complications, particularly for larger lesions ( ≥ 2 cm), which were associated with all recorded cases of recurrence, early bleeding, and perforation in our study.

 In a comparative analysis of ESD and laparoscopic-assisted colorectal cancer (LAC) surgery, ESD demonstrated shorter operative times (106 minutes vs. 206 minutes) and fewer complications (6.4% vs. 13.3%). While ESD complications were limited to perforation and bleeding, LAC was associated with surgical site infections, pelvic abscesses, anastomotic leaks, and anastomotic bleeding.^13,29^ Additionally, over 90% of patients undergoing LAC for rectal cancers below the peritoneal reflection required a stoma, further emphasizing the minimally invasive advantages of ESD, particularly for elderly patients with surgical contraindications.^17,30^ In our study, perforations were managed conservatively, reinforcing ESD’s role as a safe and effective treatment option for older patients with colorectal tumors.

 Despite its advantages, ESD remains a technically demanding procedure with inherent risks, necessitating careful patient selection, particularly for colorectal lesions. Various classification systems have been proposed to identify lesions suitable for ESD, but appropriate case selection remains challenging. Only 8-10% of superficially resected lesions are classified as invasive cancers, with the majority being benign or precancerous.^14^ This highlights the need for improved diagnostic tools and criteria to optimize patient outcomes.

 Significant disparities in ESD outcomes have been observed between Eastern and Western countries, likely due to differences in training, procedural volume, and lesion characteristics.^31^ Colorectal lesions, in particular, present unique challenges due to limited operational space, physiological curvatures, and the thin intestinal mucosa, which increases the risk of perforation.^32^ Ongoing research is focused on developing innovative techniques to enhance the safety and efficacy of ESD for colorectal lesions.

 Our findings support the use of ESD as a safe and effective technique for the complete en bloc resection of gastrointestinal lesions, with low rates of recurrence and perforation. However, careful patient selection and ongoing advancements in endoscopic technology are essential to further improve outcomes and reduce complications. Future studies should build on these findings by conducting multicenter research involving diverse patient populations and surgical teams to enhance the generalizability and robustness of the results. It would be valuable to compare the outcomes of en bloc ESD with other treatment methods, such as surgery and the Piecemeal technique, in terms of recurrence rates, complications, and long-term efficacy. Additionally, research should investigate the impact of surgeon experience and skill level on resection outcomes, particularly when employing advanced techniques such as ESD. Long-term follow-up studies are also necessary to evaluate the durability of resection outcomes, including recurrence rates and patient quality of life after treatment. Furthermore, future studies should include a broader range of patients, encompassing different types of gastrointestinal lesions, comorbidities, and demographic backgrounds, to better understand the applicability of various resection methods. Cost-effectiveness analyses could provide additional insights by comparing factors such as hospital stay duration, complication rates, and resource utilization across different techniques. Ultimately, the exploration of emerging technologies, such as robotic-assisted resection and advanced imaging techniques, may present new opportunities to enhance the precision and outcomes of gastrointestinal lesion resection. By addressing these areas, future research can contribute to the development of more effective and widely applicable treatment strategies.

## Conclusion

 The study demonstrates that ESD is a safe and effective technique for the complete en bloc resection of gastrointestinal lesions, with low rates of recurrence and perforation. The overall complete resection (R0) rate was 95.1%, with recurrence observed in only 2.5% of cases. Complications such as perforation and immediate bleeding were minimal, and no delayed bleeding was reported. These findings highlight the superiority of en bloc resection over the piecemeal method, particularly for larger lesions ( ≥ 2 cm), which were associated with higher rates of recurrence and complications. ESD offers significant advantages over surgical interventions, including shorter operative times, fewer complications, and faster recovery, making it a viable option for elderly patients or those with surgical contraindications. However, the technical complexity of ESD necessitates careful patient selection and skilled endoscopists to optimize outcomes. Future research should focus on multicenter studies, long-term follow-up, and comparisons with other treatment modalities to further validate the efficacy and safety of ESD. Additionally, advancements in endoscopic technology and training are essential to enhance the adoption and success of ESD globally.

## Limitations

 The study has several limitations that should be acknowledged. First, the selection of patients from a single center may restrict the generalizability of the findings. Practices, patient demographics, and surgeon expertise at one center may not fully represent those at other institutions, potentially introducing bias. Additionally, the study focused exclusively on patients who underwent resection of gastrointestinal lesions using the piecemeal and en bloc methods, which may not account for the diversity of lesion types, patient characteristics, or surgical techniques used in broader clinical settings. The results may also be influenced by the specific experience and techniques of the surgeons involved, which may not reflect outcomes achieved by surgeons with varying levels of expertise. Furthermore, the lack of multicenter data limits the ability to generalize the findings to different geographic regions or healthcare systems. Finally, the study did not compare the outcomes of the piecemeal and en bloc methods with other treatment modalities, such as ESD or surgery, which could provide a more comprehensive understanding of the optimal approaches for gastrointestinal lesion resection.
